# Effectiveness of Educational Technology in Promoting Quality of Life and Treatment Adherence in Hypertensive People

**DOI:** 10.1371/journal.pone.0165311

**Published:** 2016-11-16

**Authors:** Ana Célia Caetano de Souza, Thereza Maria Magalhaes Moreira, Edmar Souza de Oliveira, Anaíze Viana Bezerra de Menezes, Aline Maria Oliveira Loureiro, Camila Brasileiro de Araújo Silva, Jair Gomes Linard, Italo Lennon Sales de Almeida, Samuel Miranda Mattos, José Wicto Pereira Borges

**Affiliations:** 1 Postgraduate Program Clinical Care in Nursing and Health (PPCCLIS), Universidade Estadual do Ceará (UECE), Unit of Clinical Pharmacology, Center for Research and Development of Medicines (NPDM), Universidade Federal do Ceará, Fortaleza, Brazil; 2 Postgraduate Program Clinical Care in Nursing and Health (PPCCLIS), Postgraduate Program in Public Health (PPSAC), Universidade Estadual do Ceará, Fortaleza, Brazil; 3 Hospital Carlos Alberto Studart Gomes, Fortaleza, Brazil; 4 Universidade Estadual do Ceará (UECE), Fortaleza, Brazil; 5 Liver Transplant Unit, Hospital Geral de Fortaleza, Fortaleza, Brazil; 6 Postgraduate Program in Public Health (PPSAC), Universidade Estadual do Ceará, Fortaleza, Brazil; 7 Scholarship of scientific initiation, Universidade Estadual do Ceará, Fortaleza, Brazil; 8 Postgraduate Program Clinical Care in Nursing and Health (PPCCLIS), Universidade Estadual do Ceará, Fortaleza, Brazil, Postgraduate Program in Health and Community (PPGSC), Universidade Federal do Piauí, Teresina, Brazil; Florida International University Herbert Wertheim College of Medicine, UNITED STATES

## Abstract

The objective of this study was to test the effectiveness of an educational intervention with use of educational technology (flipchart) to promote quality of life (QOL) and treatment adherence in people with hypertension. It was an intervention study of before-and-after type conducted with 116 hypertensive people registered in Primary Health Care Units. The educational interventions were conducted using the flipchart educational technology. Quality of life was assessed through the MINICHAL (lowest score = better QOL) and the QATSH (higher score = better adherence) was used to assess the adherence to hypertension treatment. Both were measured before and after applying the intervention. In the analysis, we used the Student’s t-test for paired data. The average baseline quality of life was 11.66 ± 7.55, and 7.71 ± 5.72 two months after the intervention, showing a statistically significant reduction (p <0.001) and mean of differences of 3.95. The average baseline adherence to treatment was 98.03 ± 7.08 and 100.71 ± 6.88 two months after the intervention, which is statistically significant (p < 0.001), and mean of differences of 2.68. The conclusion was that the educational intervention using the flipchart improved the total score of quality of life in the scores of physical and mental domains, and increased adherence to hypertension treatment in people with the disease.

## Introduction

Hypertension is a multifactorial clinical condition characterized by high and sustained levels of blood pressure, when systolic pressure is ≥ 140mmHg and diastolic pressure is ≥ 90mmHg. The blood pressure can be classified as optimal, normal, high-normal, stage 1 hypertension, stage 2 hypertension, stage 3 hypertension and isolated systolic hypertension [1].

High blood pressure (hypertension) is a serious public health problem that affects a significant portion of the population. The disease prevalence among adults is 25% worldwide and the estimate for 2025 is 29% [2]. A systematic review showed that hypertension prevalence in Brazil is about 30% [3].

The control of blood pressure levels and consequently of the disease, can be effective with non-pharmacological antihypertensive treatment (in association with drugs or not), and improve the lives of hypertensive people. Despite the evidence that antihypertensive treatment significantly reduces cardiovascular morbidity and mortality, the inadequate disease control has been observed in several countries [4–7].

The treatment adherence is one of its most important and challenging aspects that stands out in the prevention and control of the disease. Worldwide, governments, health professionals, and researchers have studied and worked the aspects involving treatment adherence in hypertension [8].

An important issue related to hypertension control and treatment adherence has been the quality of life (QOL) of people with the disease, because the antihypertensive treatment impacts on their quality of life [9–11]. The QOL of hypertensive people is affected by several factors, including those linked to the chronic degenerative condition, the discovery of the disease, the deficits in physical, emotional and social aspects and those related to the drug therapy [12].

The negative impact of hypertension on people’s lives and the difficulties found with the implementation of actions or health behaviors have provided important reflections for those involved [13,14] (health professionals, government, researchers and people with the disease), aiming to think of innovative strategies for promoting control of the disease and its complications.

Thus, educational technologies emerge as a strategy in disease prevention and health promotion. New educational technologies have allowed the encounter with other individuals for the development of health promotion actions jointly [14]. These technologies are innovative and provide health practices that generate greater empowerment, with consequent advances in promoting quality of life and adherence to treatment.

The educational technology of a flipchart titled ‘Hypertension: promoting quality of life and adherence to treatment’ was built for this study and previously validated [15]. The development of the educational technology (flipchart) was based on the methodological framework called ‘Teaching patients with low literacy skills’ [16] of the Harvard University.

The objective of this study was to test the effectiveness of an educational technology (flipchart) to promote quality of life and adherence to treatment in people with hypertension.

## Material and Method

This is a quasi-experimental study of before-and-after design conducted with hypertensive people registered in health centers. The data collection period was between November 2014 and March 2015 in all Primary Health Care Units (UAPS) of a capital city located in the Northeast of Brazil. Educational interventions using the flipchart technology were applied for groups of six to eight people diagnosed with hypertension.

### Data collection instruments

The following data collection instruments were used: Questionnaire on Adherence to Systemic Hypertension Treatment (QATSH)-[17], and the Mini Quality of Life Questionnaire in Blood Hypertension (MINICHAL-BRAZIL), tested and validated and in Brazil [18]. Both questionnaires were applied before the first intervention and after the end of the last.

The QATSH is a questionnaire developed by Rodrigues [17] in his doctoral thesis entitled ‘Adherence to hypertension treatment: development of an assessment instrument based on Item Response Theory (IRT)’. This instrument consists of 12 items and evaluates the treatment adherence of hypertensive individuals by measuring it with a six-level scale ranging from 60 to 110, in which the highest level represents the greatest adherence to treatment. The MINICHAL [18] is a specific questionnaire to assess the quality of life of hypertensive people with 17 questions grouped in two areas (Mental Status and Somatic Manifestations). The Mental Status domain comprises questions 1–9 and the Somatic Manifestations domain comprises questions 10–16. Each question has four answer choices. The scores for each domain are obtained by adding the scores of the questions within each domain. The average of scores ranges from 0 to 27 for the Mental Status domain and 0–21 for the Somatic Manifestations domain. The closest to zero is the MINICHAL final score, the better the quality of life. The item 17 is not scored nor enters the sum of the scores.

### Application of educational technology "flipchart"

The educational technology used was a flipchart, which is a visual resource with pages in a logical sequence, enabling the development of a single message in a progressive and logical manner. It is used to assist in classes, lectures, demonstrations, workshops, among others [19].

The flipchart contains 11 pictures (front) and 11 script sheets (back) (S1 Fig). The pictures represent people with hypertension facing their illness in day to day and playfully discuss treatment adherence and quality of life. The script sheets standardize the presentation of the themes by indicating the content addressed by the professional at the time of discussion.

To apply the educational technology was used the educational intervention of workshops, which allow the interaction and exchange of knowledge between the health professional and patients with hypertension. This intervention was performed in three stages with application of the educational technology for the same group of hypertensive people. Each intervention lasted 60–90 minutes. On average, there was an interval of twenty-five days between every stage of the educational technology application.

The educational interventions were conducted by health care professionals (nurses and physical education teachers) and undergraduate students of nursing and physical education courses.

In the first stage, were conducted the following activities: team presentation, reading and signature of the informed consent form, explanations about the educational intervention, completion of the MINICHAL and QATSH questionnaires, verification of anthropometric and blood pressure measurements and application of the educational intervention with use of the flipchart. The first five pictures were presented according to the back of the script sheet and discussed, including an overview of hypertension.

In the second stage, the anthropometric and blood pressure measurements were recorded, and the educational intervention was performed. The pharmacological and nonpharmacological treatment was addressed with emphasis on adherence to treatment, and use of three pictures in sequel.

In the third and last stage, the MINICHAL and QATSH questionnaires were filled out again, the measurements of weight, waist circumference and blood pressure were taken, and the educational intervention with the flipchart was applied. At this time, was addressed the issue of quality of life in hypertension.

The patients with hypertension were invited for the educational interventions (first stage) by nurses and community health agents of the Primary Care Units (PCU) where they were registered. For the subsequent activities (second and third stages), patients were invited by telephone contact made by the researchers.

The sociodemographic data collected were gender, age, educational level, income and marital status. The clinical variables were hypertension time, type of treatment, systolic blood pressure, diastolic blood pressure, weight, body mass index (BMI) and waist circumference (WC).

The study participants were adults, mostly elderly. The BMI cutoffs of the nutritional status of the elderly were adopted to calculate the body mass index of participants (>27kg/m^2^ = weight excess) [20].

The sample has been designed in order to provide a power of 90% to demonstrate the benefit of the flipchart, at a significance level of 5%, based on the changing in the total score of MINICHAL questionnaire. Thus, it was established that a difference of at least 2.5 points in the value of this parameter measured at baseline and 60 days after the intervention should be detected, the clinically significant difference, considering a standard deviation of 5.7 points, according to previous study [9]. Therefore, the sample size was calculated in 110 subjects.

The inclusion criteria were people diagnosed with hypertension, aged over 18 years and registered in Primary Health Care Units (UAPS–Unidade de Atenção Primária à Saúde), and the exclusion criterion was blindness.

The IBM SPSS Statistics for Windows version 20.0 (IBM Corp., Armonk, NY, USA, 2011) was used for data processing. The mean and standard deviation statistical measures were calculated. The Kolmogorov-Smirnov (KS) normality test indicated the use of the Student’s t-test for parametric data. To compare the average quality of life and adherence to treatment before and after the flipchart application, was used the Student’s t-test for paired data.

The participants signed an informed consent form to give their permission to take part in the study. The Research Ethics Committee of the Universidade Estadual do Ceará approved the study under number 723.860 (S1 File).

## Results

The study included 116 participants diagnosed with hypertension. The majority (78.44%) was female; 67.24% were elderly, with average age of 64.58±10.87 years; 71.55% had low educational level, and over half of the sample (56.89%) lived with a partner.

The time since hypertension diagnosis varied from six months to 60 years, with a mean of 12.49 ±10 years. Regarding the types of treatment, less than half of participants (40.52%) used only the pharmacological treatment, two people (1.72%) used only the nonpharmacological treatment, and more than half (57.76%) adopted both treatments (Table 1).

**Table 1:** Sociodemographic and clinical characterization of hypertensive people who participated in the educational intervention with flipchart.

Characteristics	Ƒ	Ƒ*r*	Mean ± SD
**Gender**			
Male	25	21.55	-
Female	91	78.44	-
**Age**			
Elderly	78	67.24	64.58±10.87
Not elderly	38	32.75	
**Educational level**			
Low educational level	83	71.55	-
Medium /high educational level	33	28.44	-
**Marital status**			
With partner	66	56,89	-
No partner	50	43,10	-
**Time with hypertension**	**-**	**-**	12.49 ±10.94
**Type of treatment**			
Pharmacological only	47	40,52	**-**
Non-pharmacological only	2	1,72	-
Both	67	57,76	-
**SBP (mmHg)**	**-**	**-**	135.19 ± 19.90
**DBP (mmHg)**	**-**	**-**	75.50 ± 11.46
**Wt**	**-**	**-**	70.24 ± 14.58
**BMI**	**-**	**-**	30.22 ± 5.66
**WC (cm)**	**-**	**-**	103.74 ± 12.48

SBP, Systolic blood pressure; DBP, Diastolic blood pressure; Wt, Weight; BMI, Body Mass Index; WC, Waist circumference

In the first phase, the average systolic blood pressure (SBP) was 135.19 ± 19.90 mmHg, and the average diastolic blood pressure (DBP) was 75.50 ± 11.46 mmHg. Another variable evaluated in the study was weight, with average of 70.24 ± 14.58 Kg among participants. The average body mass index (BMI) was 30.22 ± 5.66 kg/m^2^, demonstrating weight excess, and the average waist circumference (WC) was 103.74 ± 12.48 cm.

In the third phase, the average systolic blood pressure (SBP) was 128.55 ± 20.13 mmHg, showing a statistically significant reduction (p<0.001). The average diastolic blood pressure (DBP) was 73.56 ± 10.88 mmHg, showing a reduction, though not statistically significant (p = 0.054). Two months after the flipchart intervention, the average weight was 70.41 ± 14.82 Kg (p = 0.289) and the BMI was 30.29 ± 5.78 (p = 0.294), showing that both these measures remained unmodified. The average WC was 104.88 ± 12.47 cm two months after the application of the flipchart (p = 0.009) (Table 2).

**Table 2:** Systolic and diastolic blood pressure, weight, BMI and WC before and after applying the flipchart (n = 116).

Parameter	Baseline Mean ± SD	Two months Mean ± SD	Significance^a^	MD	CI 95%
**SBP (mmHg)**	135.19 ± 19.90	128.55 ± 20.13	p<0.001	6.64	3.20 to 10.08
**DBP (mmHg)**	75.50 ± 11.46	73.56 ± 10.88	p = 0.054	1.94	-0.03 to 3.91
**Wt**	70.24 ± 14.58	70.41 ± 14.82	p = 0.289	-0.17	-0.48 to 0.15
**BMI**	30.22 ± 5.66	30.29 ± 5.78	p = 0.298	-0.07	-0.21 to 0.06
**WC (cm)**	103.70 ± 12.54	104.88 ± 12.47	p = 0.009	-1.18	-2.06 to -0.29

SD, standard deviation; MD, Mean of differences; CI 95%, confidence interval of 95% for the mean of differences; SBP, Systolic blood pressure; DBP, Diastolic blood pressure; Wt, Weight; BMI, Body Mass Index; WC, Waist circumference.

^a^ *p* values were calculated using the t-test.

**Fig 1:**
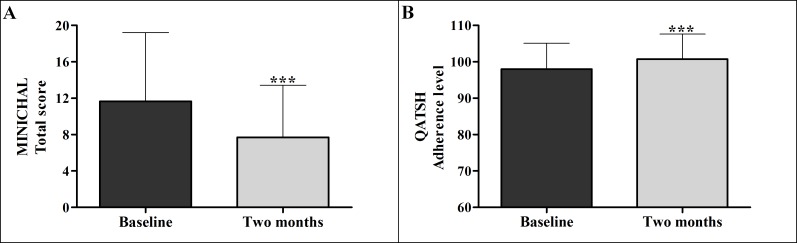
Quality of life (total score) and Adherence to treatment before and after applying the flipchart (n = 116).

The two outcome variables of the study (QOL and treatment adherence) changed significantly with use of the flipchart intervention. The average baseline quality of life was 11.66 ± 7.55 the application of the flipchart, and 7.71 ± 5.72 two months after the application of the flipchart, with a statistically significant decrease (p <0.001) and mean of differences of 3.95. The average baseline adherence to treatment was 98.03 ± 7.08 and 100.71 ± 6.88 after using the flipchart intervention, showing statistically significant increase (p < 0.001) and with mean of differences of 2.68 (Fig 1 and Table 3).

**Table 3:** Quality of life and adherence to treatment before and after applying the flipchart (n = 116).

Parameter	Baseline Mean ± SD	Two months Mean ± SD	Significance^a^	MD	CI 95%
**Quality of life**	11.66 ± 7.55	7.71 ± 5.72	p<0.001	3.95	2.78 to 5.13
**Adherence to treatment**	98.03 ± 7.08	100.71 ± 6.88	p<0.001	-2.68	-4.10 to -1.27

SD, Standard deviation; MD, Mean of differences; CI 95%, confidence interval of 95% for the mean of differences

^a^ *p* values were calculated using the t-test.

**Fig 2:**
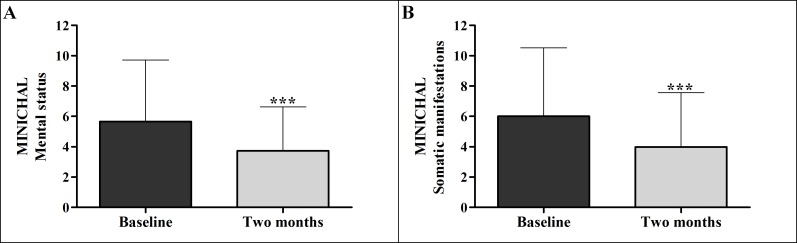
Evaluation of quality of life performed before and after exposure to the flipchart (n = 116). The Figure shows quality of life according to the domains of Mental Status and Somatic Manifestations of the MINICHAL.

Regarding the score of the Mental Status domain of the MINICHAL, the average baseline value was of 5.66 ± 4.06 and 3.72 ± 2.91 after two months application of the flipchart, with statistically significant improvement (p <0.001). For the Somatic Manifestations domain, the average score before the intervention was 6.01 ± 4.50 and 3.98± 3.58 after application of the flipchart, also with statistically significant improvement (p <0.001). The mean of differences was 1.94 in the Mental Status domain and 2.03 in the Somatic Manifestations domain (Fig 2 and Table 4).

**Table 4:** Evaluation of quality of life performed before and after exposure to the flipchart (n = 116).

Domain^a^	Baseline Mean ± SD	Two months Mean ± SD	Significance^b^	MD	CI 95%
**Mental status**	5.66 ± 4.06	3.72 ± 2.91	p<0.001	1.94	1.17 to 2.70
**Somatic manifestations**	6.01 ± 4.50	3.98 ± 3.58	p<0.001	2.03	1.37 to 2.68

SD, Standard deviation; MD, Mean of differences; CI 95%, confidence interval of 95% for the mean of differences.

^a^The table shows quality of life according to the domains of Mental Status and Somatic Manifestations of the MINICHAL.

^b^ *p* values were calculated using the t-test.

## Discussion

In this study, the educational intervention using the flipchart for the group of hypertensive people was associated with the reduction of systolic blood pressure (average decrease of 6.6 mmHg in SBP among research participants). There was also a reduction of diastolic pressure, although not statistically significant. A study conducted in Canada showed similar findings [21] on the effects of an interdisciplinary educational program in hypertension, with a reduction of SBP by 10.8 mmHg. A study conducted in Iran [22] that evaluated the care partners model for hypertension control in Iranian northern rural populations also demonstrated reductions in the mean SBP of 11.4 mmHg.

Regarding diastolic pressure, a study of educational intervention carried out in the United States [23] showed improvement in the participants’ blood pressure levels, although the DBP reduction was not statistically significant, with p = 0.058 for all participants.

Other measures (weight, BMI and WC) evaluated in the study did not show significant changes, probably given the short time of the educational intervention for hypertensive people and because those variables are more difficult to change. A study in Brazil [14] showed the need of using the educational technology as a program with longer duration than a year to observe significant changes in anthropometric and clinical parameters.

Two months after the educational intervention with flipchart, there was an increase of 3.9 points in the total score of quality of life, with improvements in the domains of Mental Status and Somatic Manifestations. The scores of the present study were higher than those found in a study conducted in Germany [9] (2.6, 1.80 and 0.80 respectively) that used the same instrument of quality of life. Other studies have also evaluated the quality of life of hypertensive people from domains or components and showed statistically significant improvements in mental and physical domains [10,11, 24].

Although there are few studies related to the role of interventions, educational activities or health education on the quality of life of hypertensive people, some have demonstrated that these actions can improve the quality of life of people with the disease, making the educational action a promoter of quality of life [22, 24].

The educational intervention also promoted statistically significant increase in treatment adherence. Within two months, patients showed greater adherence to the drug treatment. They were also more likely to adopt a diet almost without fat, sweets and sugary drinks.

The adherence to hypertension treatment is a complex issue of great importance for the public health and people with the disease. In addition, it is of hard reach because of the many aspects and factors involved, which are of economic, physical, psychological, social and cultural nature.

Thus, several studies sought to understand how treatment adherence can be improved and demonstrated that monitoring the quality of life is one of the ways to improve it with hypertensive people. Moreover, that knowledge of Health-Related Quality of Life (HRQOL) in hypertensive people and of the relationship between these two issues is a reliable determinant of cardiovascular events that can help with preventing or reducing the incidence of cardiovascular disease [7, 25].

Finally, the limitations of this study relate to the lack of a control group to observe the differences in quality of life scores and coefficient of treatment adherence between the groups. However, the educational material used in educational interventions is of low cost and easy application, and can be used by all health professionals caring for people with hypertension in the various scenarios where these people are assisted. The material also demonstrated positive impact on two important and complex constructs within the context of hypertension, which are QOL and treatment adherence.

## Conclusion

The educational intervention with the flipchart titled *Hypertension*: *promoting quality of life and adherence to treatment* improved the total score of quality of life and the scores of Somatic Manifestations and Mental Status domains. It also increased the adherence to hypertension treatment in people with the disease.

In this sense, the health-related quality of life can be affected by strategies or actions that provide important reflections in the life context of hypertensive people. Improvements in the quality of life and adherence to treatment in people with hypertension are related to the care and attention provided by health professionals. The association of educational interventions, attentive listening and the dialogue established within health facilities and in community spaces can facilitate the decision-making about the use of strategies or actions by patients in order to promote improvements in their living and health conditions.

## Supporting Information

S1 Fig Educational technology—Flipchart. (PDF)

S1 File Approval Committee of Research Ethics. (PDF)
