# Supplementary material for: Effectiveness of Educational Technology in Promoting Quality of Life and Treatment Adherence in Hypertensive People
Source: PLoS One. 2016 Nov 16;11(11):e0165311. doi: 10.1371/journal.pone.0165311 (PMC5112805; doi:10.1371/journal.pone.0165311)

Desing of Flipchart

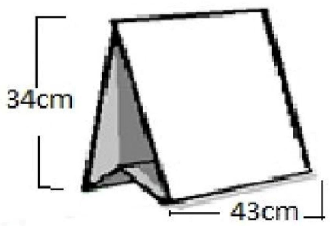

Pictures (front)	Script sheets (back)	Pictures (front)	Script sheets (back)

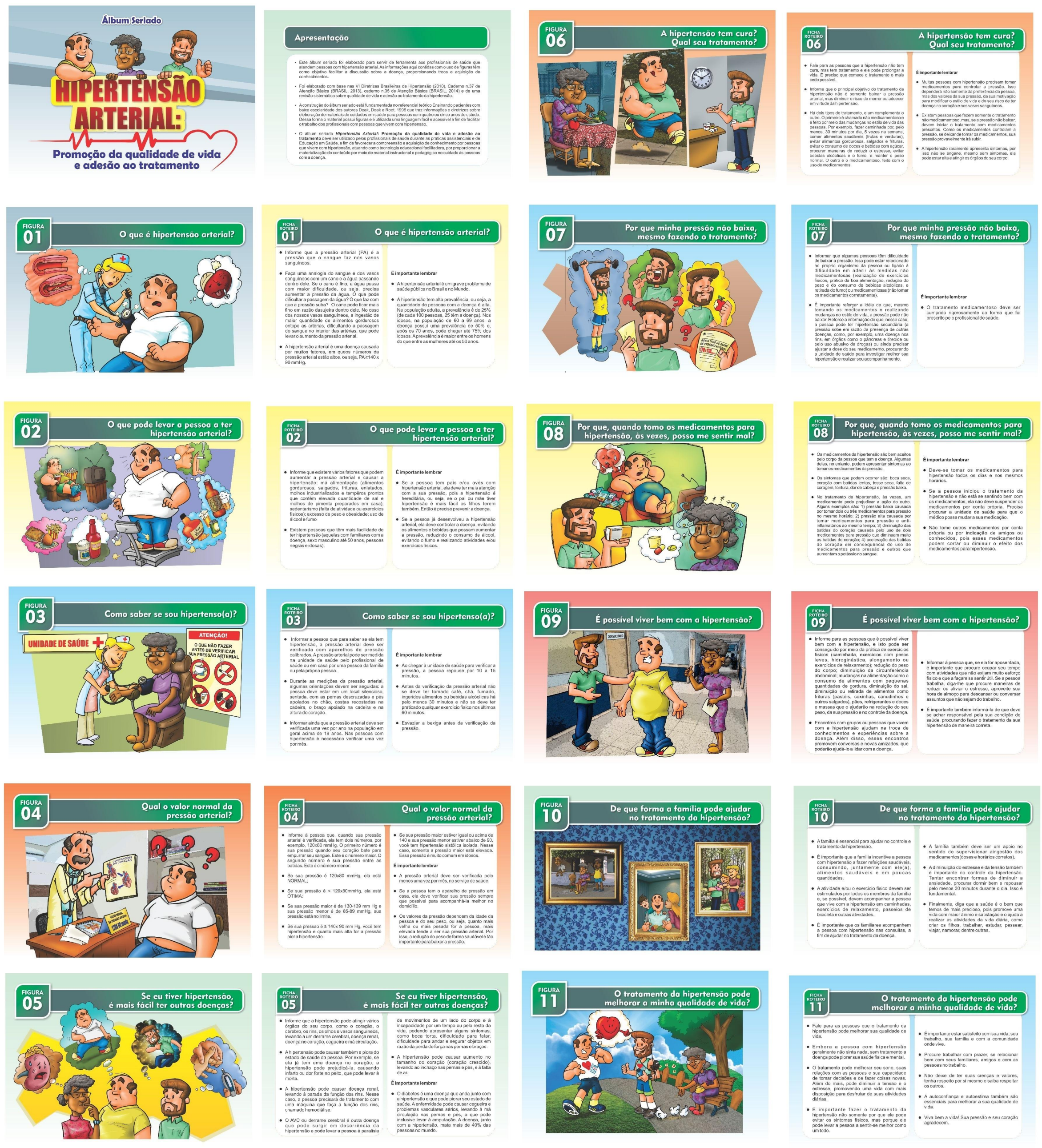

Supplement: S1 Fig — (PDF) [file pone.0165311.s001.pdf]
