# Supplementary material for: Effectiveness of Educational Technology in Promoting Quality of Life and Treatment Adherence in Hypertensive People
Source: PLoS One. 2016 Nov 16;11(11):e0165311. doi: 10.1371/journal.pone.0165311 (PMC5112805; doi:10.1371/journal.pone.0165311)
Supplement: S1 File — (PDF) [file pone.0165311.s002.pdf]

PARECER CONSUBSTANCIADO DO CEP

DADOS DO PROJETO DE PESQUISA

Título da Pesquisa: CONSTRUÇÃO E VALIDAÇÃO DE TECNOLOGIA EDUCACIONAL COMO SUBSÍDEO À AÇÃO DO ENFERMEIRO NA PROMOÇÃO DA QUALIDADE DE VIDA E ADESÃO AO TRATAMENTO DE HIPERTENSOS

Pesquisador: ANA CÉLIA CAETANO DE SOUZA

Área Temática:

Versão: 2

CAAE: 27080014.0.0000.5534

Instituição Proponente: PROGRAMA DE PÓS-GRADUAÇÃO EM ENFERMAGEM E SAÚDE

Patrocinador Principal: Financiamento Próprio

DADOS DO PARECER

Número do Parecer: 723.860

Data da Relatoria: 09/06/2014

Apresentação do Projeto:

A qualidade de vida de pacientes com hipertensão é afetada por vários fatores, dentre eles ligados à própria existência da enfermidade e seu caráter crônico-degenerativo, à descoberta da doença que leva a modificações na vida dos doentes, aos déficits nos aspectos físico, emocional e social, e aqueles relacionados à terapia medicamentosa (GUSMÃO, 2004). Uma questão relevante relacionada à importância da avaliação subjetiva do paciente é a adesão ao tratamento, pois aderir a práticas ou intervenções depende da motivação e da satisfação do indivíduo frente aos resultados alcançados pelo tratamento, sendo necessário saber se ele o avalia como eficaz, melhorando seu estado de saúde (BLAY MARCHESONI, 2013). Os pesquisadores pretendem desenvolver e validar uma tecnologia educacional que subsidie o enfermeiro na promoção da qualidade de vida e adesão ao tratamento de hipertensos. Trata-se de um estudo do tipo metodológico, quase-experimental, com abordagem quantitativa, que será desenvolvido em duas fases: Na primeira será feito um estudo metodológico para a construção e validação de um álbum seriado para promoção da qualidade de vida e adesão ao tratamento de hipertensos. A segunda fase consistirá da validação clínica, com um estudo de intervenção. A pesquisa será realizada em três postos de saúde pertencentes à Secretária de Saúde do Município de Fortaleza. A população do estudo será constituída de participantes com diagnóstico de hipertensão arterial e a amostra de

384 pessoas, calculada a partir da fórmula de população infinita. Os critérios de inclusão são: pessoas com idade igual ou maior que 18 anos, diagnóstico médico exclusivo de hipertensão com pelo menos um ano de tratamento da doença. Apresentar sequelas neurológicas constituirá critério de exclusão. Para validação do álbum seriado por especialistas, será utilizado o Índice de Validade de Conteúdo (IVC) que mede a proporção de juízes em concordância sobre determinados aspectos do instrumento e de seus itens que permite analisar cada item separadamente e o instrumento como um todo. Uma escala tipo Likert será utilizada para avaliar a concordância e a representatividade dos itens. Na validação clínica será utilizado o teste alfa de Cronbach para avaliar a consistência interna. Regressão logística será usada para analisar as relações entre as múltiplas variáveis independentes (uso da tecnologia educacional e características sócio-demográficas) e as variáveis dependentes (promoção da qualidade de vida e adesão ao tratamento anti-hipertensivo).O projeto foi delineado segundo a Resolução 466/2012 e os participantes, hipertensos e especialistas em enfermagem, assinarão um termo de consentimento livre e esclarecido.

Objetivo da Pesquisa:

Desenvolver uma tecnologia educacional que subsidie o enfermeiro na promoção da qualidade de vida e adesão ao tratamento de hipertensos, e sua validação.

Avaliação dos Riscos e Benefícios:

Riscos = Descobrir que possui baixa adesão ao tratamento e qualidade de vida reduzida.

Benefícios = Como benefícios, o retorno aos indivíduos serão: a participação de sessões de educação em saúde, discutir novas estratégias para aumentar a adesão ao tratamento e melhorar qualidade de vida.

Comentários e Considerações sobre a Pesquisa:

A proposta de pesquisa é pertinente, apresenta valor científico. Os pesquisadores esperam que seus resultados possibilitem subsidiar o enfermeiro na promoção da qualidade de vida e adesão ao tratamento de hipertensos. Entretanto, o projeto tem um custo elevado para ser financiado pelo próprio pesquisador, sem auxílio de Instituições de fomento à Pesquisa. O projeto de Pesquisa foi submetido anteriormente a este CEP e foi declarado como pendente.

Considerações sobre os Termos de apresentação obrigatória:

- Folha de Rosto, Carta de Anuência, TCLE, Cronograma, Orçamento:

Adequados e devidamente preenchidos

Recomendações:

1) Especificar o local de estudo no corpo do projeto.

Conclusões ou Pendências e Lista de Inadequações:

Nada consta.

Situação do Parecer:

Aprovado

Necessita Apreciação da CONEP:

Não

Considerações Finais a critério do CEP:

FORTALEZA, 21 de Julho de 2014

Assinado por:
Ana valeska Siebra e silva
(Coordenador)

Endereço: Av. Silas Munguba, 1700
Bairro: Itaperi **CEP:** 60.714-903
UF: CE **Município:** FORTALEZA
Telefone: (85)3101-9890 **Fax:** (85)3101-9906 **E-mail:** anavaleska@usp.br
